# Benign osteonecrosis of the external ear canal: case series

**DOI:** 10.1093/jscr/rjad016

**Published:** 2023-01-31

**Authors:** Kiyoshi Chandler, McKenna Hawthorne, Alexandros Georgolios

**Affiliations:** Poplar Bluff Medical Center, Regional Physician Services – Ear, Nose & Throat, Poplar Bluff, MO, USA; Poplar Bluff Medical Center, Regional Physician Services – Ear, Nose & Throat, Poplar Bluff, MO, USA; Poplar Bluff Medical Center, Regional Physician Services – Ear, Nose & Throat, Poplar Bluff, MO, USA

## Abstract

Benign osteonecrosis of the external ear canal is a rare pathology that commonly gets misdiagnosed as cholesteatoma of the external ear canal, keratosis obturans and malignant otitis externa. Each pathology has characteristics that allow for differentiation between them. Careful analysis is required to diagnose properly and determine the best modality of management. This case series presents two patients that were diagnosed with benign osteonecrosis of the external ear canal and is being managed conservatively with serial debridement. Response to conservative treatment has resulted in adequate control of symptoms in both patients.

## INTRODUCTION

Benign osteonecrosis of the external ear canal is a rare finding and is often confused for other pathologies, such as cholesteatoma of the external ear canal, keratosis obturans and malignant otitis externa. However, benign osteonecrosis of the external ear is pathologically different and distinct. Benign osteonecrosis is sequestration to the temporal bone, whereas keratosis obturans and external canal cholesteatoma typically occurs from destruction followed by keratin deposition [1]. The symptoms are also distinct and unique to benign osteonecrosis of the external ear canal presenting with unilateral otalgia and otorrhea with recurrent exacerbation, itching and upon otologic exam, the ear canal is patent with a healthy tympanic membrane, whereas keratosis obturans would show an obliterated ear canal. Furthermore, the sequestrum in benign osteonecrosis would need to be removed to assess the extent of damaged bone beneath [1, 2]. In this case series, the management of two patients, one of which had bilateral involvement to produce management of three ear canals that suffer from this rare disease is discussed.

## CASE SERIES

**Figure 1:**
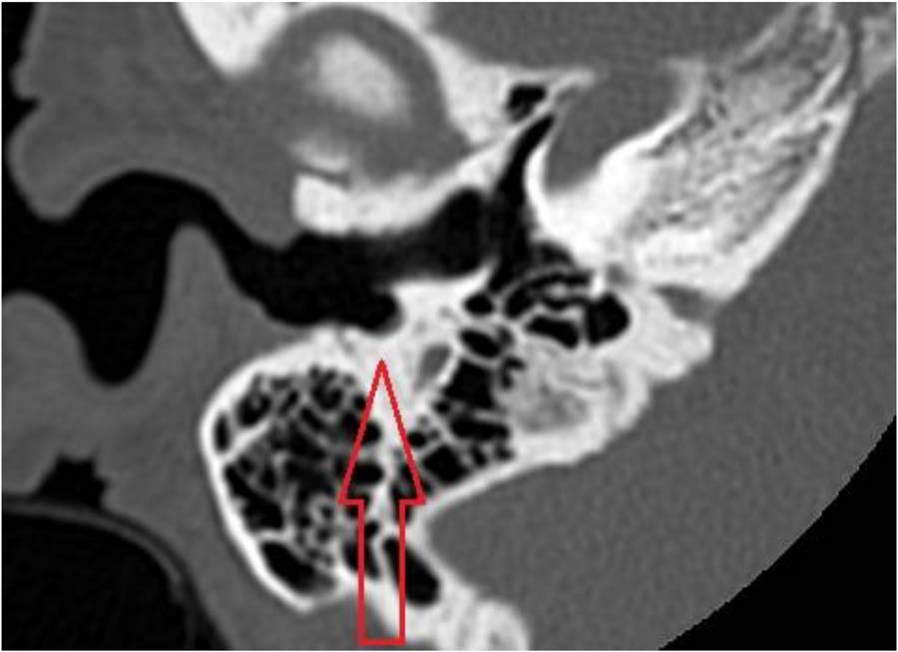
Axial cut in the inferior aspect of the external auditory canal in patient A (CT of the temporal bone) demonstrating the indentation of the inferior aspect of the external auditory canal.

**Figure 2:**
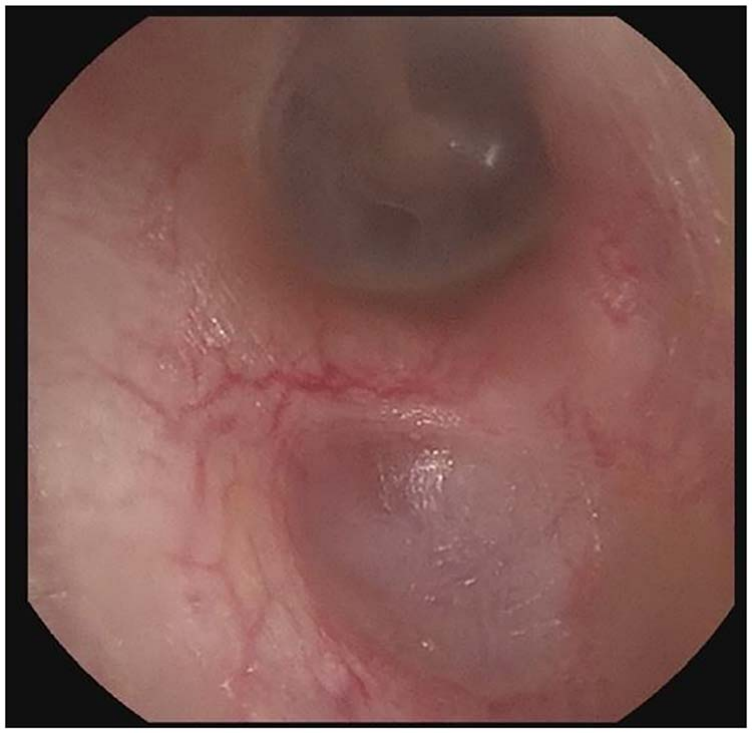
Right external auditory canal on patient A demonstrating excellent mucosalization of the indentation in the inferior aspect of the external auditory canal after serial cerumen debridement procedures in the office.

The patient evaluation and the data collection of this rare entity were performed in the senior author’s Otolaryngology practice in Poplar Bluff Regional Medical Center, Poplar Bluff, Missouri, USA (Table 1). Patient A, a 72-year-old female, was initially seen in 06/2015 and has been consistently followed for 87 months. On initial evaluation, the unusual clinical presentation triggered a workup with a CT of the temporal bone (Fig. 1), revealing a minor area of cortical erosion in the inferior wall of her right bony external auditory canal. Her initial presenting complaint was cerumen impaction on the right side associated with same side hearing loss. The patient has no history of diabetes, is treated with estradiol and progesterone tablets for hormonal replacement therapy and has no diagnosis of osteoporosis. Her blood pressure medications include atenonol and triamteren and has been on the same medications for the last seven years of our observation. The patient was managed with serial external auditory canal debridements, every 6 months. She had no history of otitis externa during the years of follow-up. Photographic documentation in her most recent visit (10/2022) revealed healthy appearing healthy mucosalization of the cavity and minimal amount of ceruminous debris (Fig. 2).

**Table 1:** Overview of Patients A and B disease courses with location, management and duration of treatment to date

Patient	Location	Gender	Age	Medications	Months of follow-up	Management
Patient A	Right ear	Female	72	Estradiol/progesterone therapy, atenolol, triamteren	87	Conservative with serial debridement q6 months
Patient B	Bilateral	Male	56	Amlodipine	35	Conservative with serial debridement q3 months

**Figure 3:**
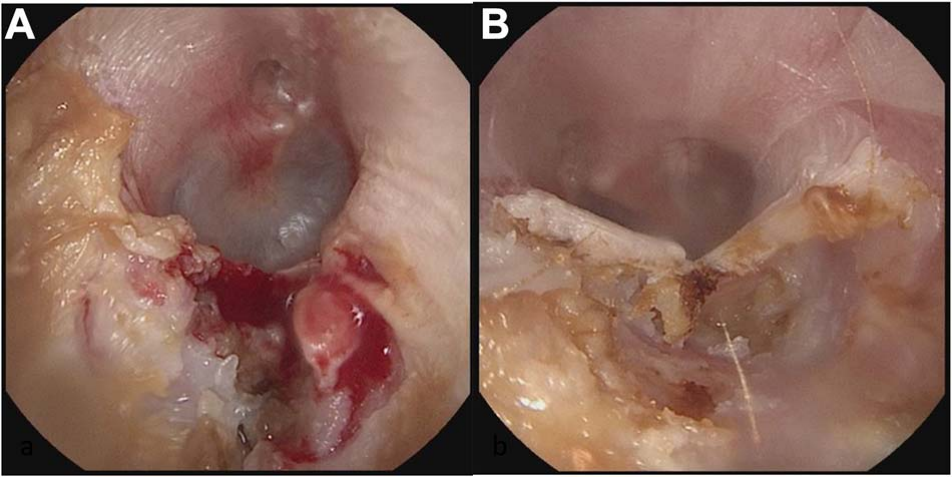
Panel **a** shows the indentation of the left external auditory canal with bony sequestration. Picture was after debridement. Panel **b** shows the right external auditory canal. Less extensive than the left, however, fragmentation and sequestration is also seen.

Patient B, a 56-year-old male, was initially diagnosed in 11/2019 and has been consistently followed for 35 months. He was also referred for cerumen impaction in the left ear canal, associated with otorrhea and hearing loss. He was diagnosed with an erosive indentation in the inferior wall of his external auditory canal, filled with ceruminous debris and minimal amount of drainage. Albeit minor, there was obvious bony sequestration in the area of the lesion and miniscule osseous fragments were retrieved from the indentation with a microsuction (Fig. 3a). He was initially followed every 6 months, but his serial debridement was eventually adjusted to every three months, due to his more severe clinical presentation. His symptoms remained mild at all times, including mild intermittent otorrhea on the left side, which was treated as it occurred, with a short course of ototopic quinolone. Interestingly, the patient was noticed to have a similar but smaller lesion (erosive indentation in the inferior wall of his external auditory canal) on the contralateral side, which was managed on the same manner with serial debridements (Fig. 3b). Patient B has also no history of diabetes or osteoporosis. He has an asymptomatic septal perforation, attributed to remote history of vasoconstrictive substances abuse. His blood pressure medications include amlodipine, also used for prolonged intervals. He has satisfactory control of his symptoms with frequent debridements, especially after his follow-up frequency was increased to every three months.

## DISCUSSION

Benign osteonecrosis of the external ear is a very rare entity with minimal cases presented in the literature. Often, benign osteonecrosis of the external ear is thought of as either keratosis obliterans or cholesteatoma of the external ear; however, benign osteonecrosis has shown to have a distinct pathology of its own. The major characteristic that differentiates benign osteonecrosis is the presence of necrotic sequestrum that does not obliterate the ear canal [1]. Presence of the sequestrum makes it difficult to appreciate the degree of necrosis of the underlying bone until the sequestrum is removed. Furthermore, Whittiker et al. identifies that there is no specific ICD-10 code making identification of prior cases difficult [3].

A common misdiagnosis includes keratosis obturans, a pathology characterized as an accumulation of keratin that results to a soft tissue plug with generalized widening of the ear, and cholesteatoma, which presents with osteonecrosis and sequestration along with ottorhea [1, 4]. Both pathologies obliterate the ear canal, which is not a characteristic present in benign osteonecrosis of the external ear. Location of the lesion also is characteristic to the pathologies, with benign osteonecrosis lesion presenting on the floor of the external canal and excluded the tympanic membrane [1].

Traditionally, bisphosphonates and diabetes has been thought to have roles in the pathogenesis of this disease, though both are more associated with malignant otitis externa. Since the nature of osteonecrosis is poor vascular supply, vascular intervention has shown to be an effective treatment [3]. Distinguishing between malignant otitis externa and benign osteonecrosis is important, as malignant otitis externa typically presents with a concurrent infection with gram negatives, commonly *Pseudomonas aeruginosa* and carries an increase for mortality and invasion into underlying structures, such as the skull base, venous systems and multiple cranial nerves. Mortality with intracranial invasion carries a high mortality rate of 20% [5].

Invasion into underlying structures is also a complication with benign osteonecrosis of the external ear. Common signs and symptoms of invasion include loud noises and conductive hearing loss with the jaw closed, indicating an invasion of the temporomandibular joint [6]. Unlike malignant otitis externa, benign osteonecrosis does not have associated pain. The common associated symptom with the benign pathology is pruritus, along with ulceration and dry sequestration [7].

Due to the similarities between these rare pathologies, a thorough work up must be conducted to rule out osteomyelitis, malignant external otitis, keratosis obturans and cholesteatoma of the external ear canal. As previously state, keratosis obturans is a keratin plug. Other symptoms would include widening of the canal, thickening of the tympanic membrane and hyperemia of the skin with granulations [8]. Bony involvement of this pathology is also diffuse along the ear canal.

Cholesteatoma of the external ear canal, another rare pathology, presents first with hearing loss that is then followed by symptoms of otalgia, otorrhea, tinnitus, pruritus, facial paralysis and headache [9]. Location of cholesteatoma was variable, with the inferior wall being the most common site though multi wall involvement was found to be the most common in one study. Invasion patterns also were variant and can invade temporal bone, mastoid bone and air cells, as well as the tympanic membrane [9].

Malignant otitis externa also has differing presentations. When compared to benign osteonecrosis of the external ear, the malignant pathology is usually associated with a bacterial infection, immunosuppression and diabetes. There are five diagnostic criteria for malignant otitis externa including persistent external otitis, granulation tissue, radiographic confirmation of osteomyelitis, cranial involvement and isolation of *P. aeruginosa* from ear drainage and three of these signs must be met in order to diagnose [9, 10]. There are no such diagnostic guidelines for benign osteonecrosis, which presents with the clinical presentation of ear fullness, decreased hearing, pain or tinnitus [3]. Furthermore, benign osteonecrosis presents with punched out ulceration with a bony, dry sequestrum, commonly found on the floor of the ear canal, whereas malignant otitis externa presents with moist granulations on the floor of the ear canal [7].

Differentiating these pathologies is important for treatment options as they differ between these diagnoses. There are also various treatment options for benign osteonecrosis of the external ear canal. Since this is a rare disease, treatment modalities are still being investigated and reported.

Due to the invasive nature of cholesteatoma, the common modality is surgical intervention and debridement, depending on the grade, whereas keratosis obturans is commonly treated conservatively with removal of the keratin plug unless there is invasion into the structures below, which then requires surgical intervention [8, 9]. Malignant otitis externa requires surgical debridement and antibiotics, particularly coverage for *P. aeruginosa*, as invasion into the underlying structures carries a high mortality risk [5].

Modalities for treatment for benign osteonecrosis of the external ear canal vary and range from surgical interventions, serial debridement and concurrent use of hyperbaric chambers. With surgical intervention, the goal is to irrigate and revascularize. One report described inverting the periosteal flap into the canal or using the temporalis or superficial temporalis fascia followed by a full-thickness skin graft [7]. The use of hyperbaric oxygen therapy was employed in other studies and found a rise in partial oxygen tension, affecting gram negative bacteria activity, as well as increasing neovascularization [2, 10]. Each of these modalities were found to be effective, but to our hands, conservative management with frequent serial debridements are adequate to control symptoms for most of the cases as evidenced by the decreased amount of ceruminous debris and healthy mucosalization in Patient A.

The interesting course of Patient B included the beginning of the disease course occurring in the right ear. Debridement was initiated, and after several years of follow-up and debridement, Patient B developed contralateral occurrence. Upon investigation, there were no changes to the patient’s medications or new diagnoses associated with this finding. Trauma has been associated to benign osteonecrosis of the external ear canal disease course, due to injury of the periosteal layer and hampering of blood supply; therefore, q-tip use was asked about and patient had denied usage [2]. Serial debridement of Patient B showed improvement of symptomatic control bilaterally.

## CONCLUSION

Benign osteonecrosis of the external ear canal is a rare pathology of the ear that is often mistaken for other pathologies, such as keratosis obturans, cholesteatoma of the external ear and malignant otitis externa. Due to the location and the subtle characteristic differences between these pathologies, misdiagnoses can occur. It is important to carefully discern between them; however, as the treatment modalities and indications have different criteria based on the diagnosis. With the diagnosis of benign osteonecrosis of the external ear canal, serial debridement was found to be an adequate treatment option to control the symptoms of the patients affected.
